# The Engram’s Dark Horse: How Interneurons Regulate State-Dependent Memory Processing and Plasticity

**DOI:** 10.3389/fncir.2021.750541

**Published:** 2021-09-13

**Authors:** Frank Raven, Sara J. Aton

**Affiliations:** Department of Molecular, Cellular, and Developmental Biology, College of Literature, Sciences, and the Arts, University of Michigan, Ann Arbor, MI, United States

**Keywords:** hippocampus, neocortical circuits, oscillations, sleep, interneurons, replay, neuronal reactivation

## Abstract

Brain states such as arousal and sleep play critical roles in memory encoding, storage, and recall. Recent studies have highlighted the role of engram neurons–populations of neurons activated during learning–in subsequent memory consolidation and recall. These engram populations are generally assumed to be glutamatergic, and the vast majority of data regarding the function of engram neurons have focused on glutamatergic pyramidal or granule cell populations in either the hippocampus, amygdala, or neocortex. Recent data suggest that sleep and wake states differentially regulate the activity and temporal dynamics of engram neurons. Two potential mechanisms for this regulation are either via direct regulation of glutamatergic engram neuron excitability and firing, or via state-dependent effects on interneuron populations–which in turn modulate the activity of glutamatergic engram neurons. Here, we will discuss recent findings related to the roles of interneurons in state-regulated memory processes and synaptic plasticity, and the potential therapeutic implications of understanding these mechanisms.

## Introduction–Engram Neurons in Context

Animals have the fundamental ability to encode, store, and retrieve information about the world around them, which is crucial for their survival. Initial memory formation is thought to rely on the activation of neurons across multiple brain regions. This set of neurons–activated during experience and reactivated upon recall–is commonly referred to as the memory trace or engram. Though the idea of the engram was postulated about a century ago (Semon, 1921), it is only over the past decade that engram neuron populations have been identified by researchers and their function manipulated, due to recent technological advances. The first studies of this kind focused on neuron populations encoding contextual information in the hippocampus, a brain structure in the medial temporal lobe which is crucial for long-term spatial and episodic memory storage. Input to the hippocampus from multiple neocortical structures, including sensory cortices, flows via the entorhinal cortex perforant pathway into the dentate gyrus (DG). Information from DG (typically encoded by a sparse granule cell neuron population) is relayed via mossy fibers to hippocampal subarea cornu ammonis 3 (CA3); CA3 pyramidal neurons project via the Schaffer collaterals to hippocampal area CA1; CA1 pyramidal neurons project to the hippocampal subiculum (and to amygdala and entorhinal cortex); subicular pyramidal neurons provide the major output to the entorhinal cortex and other neocortical structures (as well as subcortical structures such as the amygdala). This relatively simple feedforward excitatory circuit is capable of encoding and at least transiently storing a vast amount of information related to space, sensation, and sequence of events. Critically, however, each substructure has recurrent connections, and reciprocal communication between hippocampus and neocortex–which may lead to information elaboration, interaction, or modification over time (Nadel et al., 2007; Dudai, 2012; Moscovitch et al., 2016; Barron et al., 2017; Hardt and Sossin, 2020). Principal neurons (DG granule cells and CA1/CA3/subiculum pyramidal cells) in the hippocampus, as well as the neocortex, are known to be activated during new experiences, such as those leading to *de novo* associative learning. This phenomenon is easily observed using immediate-early gene (IEG) expression (e.g., *Arc*, *Cfos*, or *Npas4*), as a readout measure. Principal neurons in both neocortex and hippocampus also undergo ultrastructural and intracellular molecular changes in the hours to days following learning (O’Malley et al., 1998; Trabalza et al., 2012; Yang and Gan, 2012; Alberini and Kandel, 2014; Lu and Zou, 2017; Sliwinski et al., 2020). These changes have downstream effects on the strength of synaptic connections between neurons (Whitlock et al., 2006; Cooke and Bear, 2010, 2014), neuronal activity (Thompson et al., 1996; Ognjanovski et al., 2014, 2017; Durkin and Aton, 2016; Clawson et al., 2021), and biosynthetic/metabolic changes (Im et al., 2009; Koberstein et al., 2018; Rao-Ruiz et al., 2019).

Recently developed genetic tools have allowed experimental access to the engram neuron populations that are selectively activated during specific learning events. Multiple intersectional genetic strategies have been developed to induce recombination in activated neurons, all of which are based on transgene expression from an IEG (*Arc* or *Cfos*) promoter (Reijmers et al., 2007; Liu et al., 2012; Guenthner et al., 2013). The first studies investigating the function of engram neurons used these tools to either identify or chemogenetically or optogenetically manipulate DG engram neurons encoding specific environmental contexts. The authors of these studies expressed channelrhodopsin (ChR2) in engram cells active during contextual fear conditioning (CFC) (placement in a novel context + delivery of a foot shock). They were then able to elicit “recall” of contextual fear memory (CFM), in which mice exhibited freezing responses to hippocampal light delivery, at a later time point–even when they were in a completely dissimilar context (Liu et al., 2012). In a related study, the authors found that by pairing a shock in one context with simultaneous optogenetic activation of engram neurons tagged to express ChR2 in a second, dissimilar context, they were able to generate a false associative memory of foot shock with the context used to induce recombination (Ramirez et al., 2013). These findings suggest that experimental activation of engram neuron populations is sufficient to evoke recall of specific memories (be they true or false). More recently, engram populations have been identified and manipulated in the neocortex as well (Marshel et al., 2019; Clawson et al., 2021); activation of these neocortical engram cells has a similar effect, of re-evoking experiences occurring during their genetic tagging.

Because the majority of engram neurons identified in these studies appear to be excitatory (e.g., having the morphology of neocortical pyramidal cells, DG granule cells), one would be forgiven for concluding that memory encoding, consolidation, and recall are the domain of glutamatergic circuits. However, GABAergic interneurons–the main source of inhibition in the hippocampus and neocortex–also play a critical role in mnemonic processing. Indeed, one of the IEGs described above, *Npas4*, which is transcribed in response to learning experiences, is known to play different roles in GABAergic and glutamatergic neurons, resulting in alterations in both excitatory-to-inhibitory and inhibitory-to-excitatory neuronal connectivity (Spiegel et al., 2014). A more recent study suggests that distinct engram populations exist (for example, among DG granule cells activated during CFC), which show heightened expression of either the IEG *Cfos* or *Npas4* after CFC–but not both (Sun et al., 2020). Intriguingly, these populations differ in regard to their inhibitory input–with *Npas4*-expressing engram neurons having comparatively higher inhibitory drive. An even more interesting feature of these DG populations is that they may play different roles in CFM recall. The authors of this study found that the *Npas*-expressing engram population (which received greater inhibition) was more active when mice were discriminating between the CFM context and a similar context during recall. They also found that chemogenetic suppression of activity in the *Npas*-expressing population (but not the *Cfos*-expressing population), disrupted context discrimination. Conversely, inhibition of the Cfos-expressing engram population (which received less inhibition) increased contextual fear discrimination, and activation of this population increased contextual fear generalization (Sun et al., 2020). These findings beg the question of how changes in connections between excitatory and inhibitory neurons affect the nature of, and activity among, neurons representing specific memories.

GABAergic interneurons represent about 15–20% of total neurons in hippocampus and neocortex, and are highly heterogeneous. Various interneuron types have been classified based on anatomical location, structural morphology and biochemical properties (Pelkey et al., 2017; Booker and Vida, 2018; Lourenco et al., 2020). Within both the hippocampus and neocortex, broad subclasses have been identified based on biomarker expression: those that express parvalbumin (PV+), those that express somatostatin (SST+), those that express vasoactive intestinal peptide (VIP+), and those that express cholecystokinin (CCK+). Note each of these have different subtypes, some of which are further delineated based on gene expression patterns. For example, some SST+ interneurons co-express neuropeptide Y (NPY), and some NPY+ interneurons co-express neuronal nitric oxide synthase (nNOS). Some VIP+ interneurons are also CCK+. While the functions of many of these specific subclasses are still understudied, there is significant recent data to suggest that PV+ and SST+ interneurons make major contributions to memory encoding and storage. Here we will discuss how interneurons contribute to the process of memory encoding and storage in the brain, to shape the engram. Because there is a growing appreciation of the roles of sleep states in promoting memory storage, and their roles more broadly in regulating inhibitory transmission in the brain (Puentes-Mestril and Aton, 2017; Puentes-Mestril et al., 2019), we will focus our discussion on sleep-dependent consolidation mechanisms. We will describe recent evidence which indicates differential roles for interneuron function in brain states–wake, non-rapid eye movement (NREM) sleep, and rapid eye movement (REM) sleep–in regulation of memory processing.

## PV+ and SST+ Interneurons Are Critical Regulators of Memory Encoding, Storage, and Recall

**FIGURE 1:**
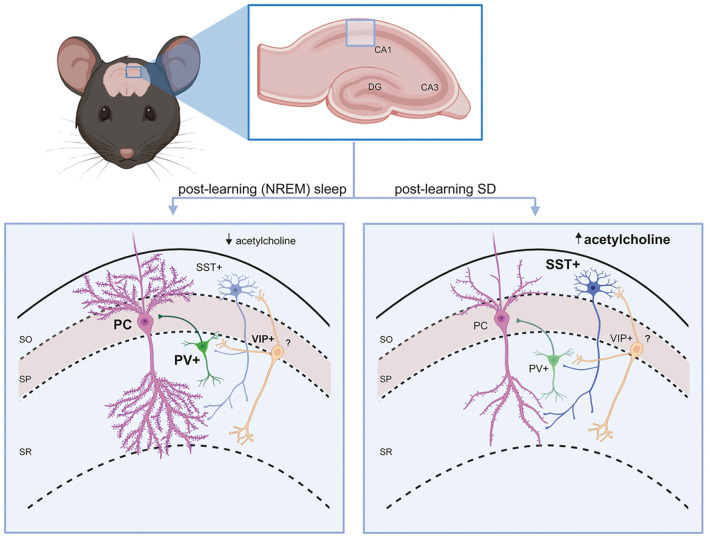
Interneuron circuit motifs and their regulation by state. A conserved interneuron motif (present throughout hippocampus and neocortex) is shown in the mouse CA1. PV+ and SST+ interneurons provide inhibitory GABAergic input to principal (glutamatergic) cells’ (PCs’) soma/axon initial segment and dendrites, respectively. VIP+ interneurons inhibit SST+ interneurons in the circuit. Left: During a period of post-learning sleep, principal cells and PV+ interneurons generally become more active (Ognjanovski et al., 2014), while SST+ interneurons are relatively quiescent (due to lower acetylcholine-mediated activation in NREM sleep) (Delorme et al., 2021). Some principal neurons (engram neurons) will become selectively more active, and may form new and/or stronger synaptic connections (Clawson et al., 2021). PV+ interneurons promote more coherent NREM and REM sleep oscillations (Ognjanovski et al., 2017, 2018). All these features are essential components of sleep-dependent memory consolidation. Right: When learning is followed by SD, SST+ interneurons shown increased activity in response to higher acetylcholine levels (Delorme et al., 2021). VIP+ interneurons may also have reduced activation, reducing inhibitory input to SST+ interneurons. This leads to suppression of firing in surrounding principal neurons (Delorme et al., 2021). As a result, dendritic spines on engram neurons (and other principal neurons) will be reduced and connections between them will be weakened (Havekes et al., 2016; Raven et al., 2019; Havekes and Aton, 2020), which impairs memory consolidation.

As mentioned above, a large proportion of GABAergic interneurons in both the neocortex and hippocampus are either PV+ or SST+. These two interneuron populations both provide strong inhibition to neighboring excitatory (e.g., pyramidal or granule) neurons’ cell bodies/axon initial segments and dendrites, respectively (Booker and Vida, 2018; Lourenco et al., 2020). As discussed in more detail below, both populations receive inhibitory input from VIP+ interneurons (a subclass of selectively interneuron-targeting GABAergic neurons). This basic microcircuit motif recurs throughout the hippocampus and neocortical layers (Figure 1).

Parvalbumin interneurons constitute the largest interneuron subpopulation in the forebrain, representing roughly 40 and 25% of all GABAergic interneurons in the neocortex and hippocampal CA1, respectively (Rudy et al., 2011; Bezaire and Soltesz, 2013). Each of these interneurons typically innervate a large number of neighboring principal neurons’ perisomatic regions, exerting strong control over their firing, and contribute to both feedforward and feedback inhibition (Udakis et al., 2020). Within the hippocampus, typically fast-spiking (FS) PV+ interneurons are implicated in a number of memory processes. PV+ interneurons in CA1 and CA3 are activated following CFC (Donato et al., 2013; Ognjanovski et al., 2014, 2017; Xia et al., 2017). Disruption of CA1 PV+ interneurons’ output via targeted expression of tetanus toxin light chain leads to deficits in hippocampus-dependent spatial working memory, but leaves cortically mediated reference memory unaffected (Murray et al., 2011). As shown more recently by our lab and others, post-CFC chemogenetic or optogenetic inhibition of PV+ interneurons [in either dorsal hippocampus or medial prefrontal cortex (mPFC)] impairs CFM consolidation (Ognjanovski et al., 2017, 2018; Xia et al., 2017). A number of studies have characterized intracellular signaling pathways that must be activated within PV+ interneurons in order to support memory storage. These pathways include those known to be required for Hebbian synaptic plasticity mechanisms [i.e., long-term potentiation (LTP) and LTD]. Cell type-specific ablation of *N*-methyl-D-aspartate (NMDA) receptors in PV+ interneurons disturbs working, short-term, and long-term spatial memory (Korotkova et al., 2010). PV+ interneuron targeted knockout of γCaMKII leads to impaired consolidation of both hippocampus-dependent CFM and inhibitory avoidance, but critically, preserves consolidation of hippocampus-independent tone-cued fear memory (He et al., 2021). PV+ interneuron-targeted knockdown of brain-derived neurotrophic factor (BDNF) receptor TrkB disrupts short-term spatial working memory–although this phenotype is seen only in male mice (Grech et al., 2019). CFM consolidation relies on activity of D1/5 dopamine receptors, and downstream activation of cAMP and ERK, in hippocampal PV+ interneurons in the hours following CFC (Karunakaran et al., 2016). PV+ interneurons in various brain structures, including the insular cortex, medial septum, and nucleus accumbens also play critical roles during memory recall–coordinating activity within limbic structures during recall of appetitive and aversive associations (Trouche et al., 2019; Sans-Dublanc et al., 2020; Yiannakas et al., 2021). Altogether, these data highlight the importance of PV+ interneurons for memory processing, and underscore that the connectivity between PV+ interneurons and the principal cells they target is crucial for engram formation.

Somatostatin interneurons comprise the second-largest population of forebrain GABAergic neurons (Rudy et al., 2011; Bezaire and Soltesz, 2013). Somatostatin (a.k.a., growth hormone-inhibiting hormone or somatotropin release inhibitory factor) is released along with GABA in this diverse population of interneurons (Pelkey et al., 2017; Booker and Vida, 2018; Honore et al., 2021) and activates Gi/o-coupled receptors (Gunther et al., 2018). Axons of SST+ interneurons target the dendritic compartments neighboring principal neurons, providing a source of strong inhibition. Like PV+ interneurons, SST+ interneurons have been implicated in regulating hippocampus-dependent memory processes. However, based on the available data, it seems likely that the two populations play distinct roles in memory encoding and consolidation. For example, chemogenetic inhibition of SST+ interneurons in the DG during CFC improves CFM recall 24 h or even a full week later (Stefanelli et al., 2016). Chemogenetic activation of SST+ interneurons during CFC, in contrast, has no effect on CFM recall at 24 h post-learning, but disrupts remote CFM recall 1 week later. This bidirectional modulation of memory retention is mirrored by DG granule cell activation during remote recall; mice that are SST+ interneuron-inhibited or -activated during encoding have larger and smaller DG granule cell populations, respectively, activated during recall. Intriguingly, the same manipulations of DG PV+ interneurons’ activity are without effect on either behavioral recall or the DG network activation at recall (Stefanelli et al., 2016). In this same study, the authors found that DG SST+ interneurons, but not PV+ interneurons, are highly activated during initial memory encoding (e.g., exploring a novel context). This latter finding has recently been replicated in the prefrontal cortex, where glutamatergic synapses onto SST+ interneurons are actually potentiated by auditory-cued fear conditioning (Cummings and Clem, 2020). Based on SST+ interneurons’ activation by activity in surrounding granule cells, and the effects of acute manipulations of their activity on granule cells, the authors concluded that these neurons support lateral inhibition between granule cells in the context of hippocampal memory encoding.

Consistent with the conclusions of the study described above, several pieces of recent data suggest that hippocampal SST+ interneurons may play a critical role in precisely encoding memories. For example, a recent study showed that optogenetic inhibition of DG SST+ interneurons during training on either a contextual or object-location discrimination task led to deficits at testing 24 h later, but only if the two scenarios being discriminated were highly similar (Morales et al., 2021). Another recent study tested the role of DG SST+ interneurons in the context of encoding foreground contextual fear, where context is the most salient predictor of a foot shock, vs. background contextual fear, where foot shocks are immediately preceded by a tone cue, making context itself less salient (Raza et al., 2017). Generally, freezing responses to the conditioning context itself are reduced when mice are background conditioned, relative to foreground conditioned mice. The authors found that training-targeted chemogenetic inactivation of DG SST+ interneurons increased the activation of surrounding DG granule cells during background conditioning, and selectively increased freezing responses to context reexposure 24 h after background conditioning (Raza et al., 2017). The studies described above suggest that SST+ interneuron activation during memory encoding effectively gates the activation of surrounding excitatory neurons. This constraining of the engram population size can limit the strength of encoding for simple associations, or in some instances, can improve memory precision and prevent generalization.

The critical role for SST+ interneurons during encoding begs the question of how these interneurons contribute to subsequent consolidation. Available data suggests that cellular pathways involved in Hebbian plasticity are engaged in SST+ interneurons by learning. For example, SST+ interneuron targeted disruption of either eIF2 or mTOR (key regulators of neuronal activity-regulated translation required for structural plasticity) leads to impaired consolidation of multiple hippocampus-dependent forms of memory (Artinian et al., 2019; Sharma et al., 2020). A recent study from our lab (described in greater detail below) assessed the effects of chemogenetically activating or inhibiting DG SST+ interneurons over the hours following CFC (Delorme et al., 2021). We found that these manipulations were sufficient to disrupt or enhance CFM consolidation, as evidenced by decreased or increased contextual freezing responses, respectively, 24 h after training. Consistent with other recent findings on the function of SST+ interneurons (described above), we found that chemogenetic activation or inhibition during the consolidation phase reduced or increased cFos expression among DG granule cells, respectively. These data suggest that inhibitory gating of activity in the DG network (and the engram neuron population) by SST+ interneurons in the hours following learning constrains hippocampal memory consolidation.

This interpretation of our data is consistent with recent neuroanatomical findings from mouse neocortex in the context of consolidation of a recently learned motor task (Chen et al., 2015). The authors found that immediately following training on the task (i.e., during consolidation), axon terminals from SST+ interneurons onto distal dendrites of surrounding pyramidal neurons are gradually reduced. This same behavior was not seen for perisomatic inhibitory synapses from PV+ interneurons, which were generally increased, rather than decreased, as a function of motor training. Thus, inhibitory gating by SST+ interneurons may serve as a constraint to consolidation even outside of the hippocampus.

## State-Dependent Regulation of Interneuron Activity During Memory Consolidation

Sleep is vital to hippocampal and neocortical memory processing (Diekelmann and Born, 2010; Rasch and Born, 2013; Puentes-Mestril and Aton, 2017; Puentes-Mestril et al., 2019), with experimental sleep deprivation (SD) capable of disrupting encoding, consolidation, and recall (Yoo et al., 2007; Heckman et al., 2020). In mice, as little as a few hours of SD following learning profoundly disrupts consolidation for hippocampal-mediated spatial and contextual tasks, e.g., object-location memory and CFM (Graves et al., 2003; Vecsey et al., 2009; Prince et al., 2014; Havekes et al., 2016; Ognjanovski et al., 2018). This disruption is associated with reductions in neurons’ dendritic spine density (Havekes et al., 2016; Raven et al., 2018; Havekes and Aton, 2020), protein synthesis (Seibt et al., 2012; Tudor et al., 2016), and intracellular signaling in pathways required for synaptic potentiation (Aton et al., 2009b; Vecsey et al., 2009; Bridi et al., 2015; Dumoulin et al., 2015). Our lab has recently demonstrated that SD also profoundly disrupts principal neuron activity in the hippocampus. We found that SD decreases neuronal activity-driven phosphorylation of ribosomal protein S6 throughout the dorsal hippocampus, suggesting decreased neuronal activity (Delorme et al., 2021). This is consistent with previous findings from our lab, where IEG *Arc*’s messenger ribonucleic acid (mRNA) and protein expression is reduced in DG after a period of SD (Delorme et al., 2019). Together these findings are consistent with human brain imaging studies, which have shown that learning-associated activation of the hippocampus is disrupted by prior overnight SD (Yoo et al., 2007). In our study, we found that while CFC itself causes subsequent increases in S6 phosphorylation throughout the hippocampus (consistent with electrophysiological findings from our lab, see below) (Ognjanovski et al., 2014), SD in the hours following CFC reduces phosphorylation of S6 (Delorme et al., 2021). Taken together, these data suggest that hippocampal neurons’ activity is dramatically suppressed by SD.

To clarify which hippocampal neurons remain active in the face of SD, we next used an unbiased bioinformatics-guided approach to profile mRNAs differentially associated with ribosomes containing phosphorylated S6 (i.e., those from the most active neurons) after sleep or SD. mRNAs which were associated with phosphorylated S6-containing ribosomes were sequenced from hippocampi of mice allowed *ad lib* sleep, or subjected to 3-h SD. Using weighted gene co-expression network analysis (WGCNA), we identified known clusters of co-regulated transcripts with abundance that varied in the hippocampus as a function of prior sleep and wake amounts. We found that two clusters of known co-regulated mRNAs are upregulated together on phosphorylated ribosomes, in proportion to prior wake time. We next compared these wake-driven, clustered transcripts with previously described cell type-specific transcript profiles, using cell type-specific enrichment analysis (CSEA). We found that mRNA markers of SST+ and NPY+ interneurons (but not VIP+ or PV+ interneurons) are selectively increased after SD (Figure 1), as are transcripts associated with cholinergic and orexinergic neurons. Thus, while SD appears to reduce activity of principal neurons (as mentioned above), it increases S6 phosphorylation in SST+ interneurons. These findings are consistent with a mechanism whereby during SD, strong inhibition from SST+ interneurons suppress activity in neighboring principal neurons. We then tested the functional significance of this mechanism for CFM consolidation, by chemogenetically activating or inhibiting SST+ interneurons within DG after CFC. We find that post-CFC suppression of activity of hippocampal SST+ interneurons (mimicking changes seen with post-CFC sleep) greatly enhances sleep-dependent CFM consolidation (Delorme et al., 2021). Conversely, post-CFC activation of SST+ interneurons (mimicking changes seen with post-CFC SD) in freely sleeping mice disrupts CFM consolidation in a manner similar to SD itself. These data suggest a mechanism whereby DG SST+ interneurons (and possibly interneurons in other subregions) act as a state-dependent gate on memory consolidation, which, when activated by SD, suppresses hippocampal network activity.

An unanswered question is how sleep and SD differentially regulate the activity level of hippocampal SST+ interneurons. Our bioinformatics analysis indicated that cholinergic and orexinergic inputs to the hippocampus are more active after a period of SD vs. *ad lib* sleep (Delorme et al., 2021). Recent evidence suggests that acetylcholine has the ability to selectively activate SST+ interneurons in the hippocampus (Lovett-Barron et al., 2014; Raza et al., 2017); this selective sensitivity is mediated by both nicotinic and muscarinic receptors expressed preferentially on the neurons (Hajos et al., 1998; Jia et al., 2010; Nichol et al., 2018). Acetylcholine release in the hippocampus by medial septal inputs is known to be higher during wake vs. NREM sleep (with release increasing again during REM sleep) (Kametani and Kawamura, 1990; Teles-Grilo Ruivo et al., 2017). Based on this evidence, we tested whether gating of the hippocampal network, and CFM consolidation, are affected by manipulations of cholinergic input. We found that similar to the results of chemogenetic manipulation of SST+ interneuron activity, chemogenetic suppression of medial septal cholinergic neurons after CFC resulted in improved sleep-dependent memory consolidation and greater DG network activity. Chemogenetic activation of cholinergic inputs resulted in suppression of DG network activity and impaired memory consolidation (Delorme et al., 2021). These findings are strikingly similar to behavioral findings in human subjects, indicating that reductions in forebrain acetylcholine release are an essential component of sleep-dependent memory consolidation (Gais and Born, 2004; Rasch et al., 2006). Thus, we conclude that cholinergic activation of the hippocampal SST+ network is a major driver of memory consolidation deficits caused by sleep loss.

One caveat to this interpretation, alluded to above, is that cholinergic input to the hippocampus is typically low during NREM sleep, but is elevated (to levels similar to those seen wake, or even higher) during REM sleep (Kametani and Kawamura, 1990; Teles-Grilo Ruivo et al., 2017). While both REM and NREM sleep have been implicated in memory processing (Rasch and Born, 2013; Puentes-Mestril and Aton, 2017; Puentes-Mestril et al., 2019), the relative contribution of the two sleep states to hippocampally mediated memory consolidation is still largely unknown. As described in more detail below, recently developed genetic tools are allowing a new understanding of the respective roles of REM and NREM sleep in memory processing.

## Role of Reactivation/Replay in Sleep-Dependent Consolidation

Reactivation of neurons active during prior experience (in the hippocampus and other structures such as the neocortex) has been proposed as an essential component of systems-level memory consolidation (O’Neill et al., 2010). Such reactivation, and sequential “replay” of neuronal activity in populations of neurons activated sequentially during prior experiences, occur during offline states such as quiet wake and sleep.

The function of reactivation/replay in memory storage (and as a mediator of sleep-dependent memory storage) has been a matter of debate (Puentes-Mestril and Aton, 2017; Puentes-Mestril et al., 2019; Findlay et al., 2020). A major criticism of sequential replay is that the time course over which it is typically observed during sleep does not match the time course of memory consolidation. For example, sequential replay of hippocampal place cell activation patterns is frequently reported following running of a familiar maze–i.e., only after having run the maze daily for several days/weeks. The occurrence of replay sequences outlasts the behavior itself by only a few tens of minutes (e.g., only over the first few minutes of post-running NREM sleep). Clearly, such instances of sequential replay cannot reflect the process of consolidating newly encoded information. However, more recent work from our lab (Ognjanovski et al., 2014, 2017, 2018) and others (Giri et al., 2018) has demonstrated (using novel metrics) (Wu et al., 2018) that major changes to functional communication patterns between neurons are initiated in the hippocampal network by single-trial learning, and persist for many hours during post-learning sleep. As discussed below, these changes produce highly reliable spike timing relationships between pairs of neurons in the network–an ideal scenario for promoting spike timing-dependent plasticity in the hippocampal network during consolidation. Critically, the duration of these changes–hours (Giri et al., 2018) up to a day and possibly longer (Ognjanovski et al., 2014, 2017, 2018)–is also more compatible with a role in long-term memory consolidation, which is disrupted by interference with either sleep or hippocampal activity patterns several hours after memory encoding (Puentes-Mestril and Aton, 2017; Puentes-Mestril et al., 2019).

While the role of hippocampal and neocortical experience-encoding neurons in memory recall or perception has been well described (Liu et al., 2012; Ramirez et al., 2013; Marshel et al., 2019), very little is known about their role in memory consolidation. However, a recent study from our lab has demonstrated that in the sensory cortex, sleep-dependent reactivation of neurons activated by learning is essential for memory consolidation (Clawson et al., 2021). In this study, we demonstrated that sleep-associated reactivation of stimulus-selective neurons in primary visual cortex (V1), over the first few hours following visually-cued fear conditioning, is essential for the sleep-dependent consolidation of visually-cued fear memory. This type of memory is encoded by repeatedly pairing a visual stimulus with a foot shock, and its consolidation is disrupted by post-conditioning SD (Clawson et al., 2021). To clarify the role of sleep-associated engram reactivation in this consolidation process, neurons in V1 that were activated during presentation of the same specific visual stimulus later used as an aversive cue were genetically targeted via targeted recombination in activated populations (TRAPs) (Guenthner et al., 2013). In the transgenic mice used for TRAP in this specific study, the *cfos* promoter drives expression of an estrogen receptor-fused CRE recombinase. Using this genetic tool, CRE-dependent expression of fluorescent proteins or opsins was targeted in sensory engram neurons in V1, by presenting the visual stimulus in close temporal proximity to administration of tamoxifen. After genetic targeting, these same neurons were activated in V1 in the context of fear conditioning, using the same visual stimulus as a repeated cue for foot shock. By expressing a fluorescent protein in these “visual engram” neurons and characterizing IEG expression in V1 following conditioning, we found that engram neurons are selectively reactivated during sleep-dependent consolidation of visually-cued fear memory. To test the functional role of this reactivation, we expressed the inhibitory opsin archaerhodopsin in the V1 engram neuron population. Inhibition of engram neurons in V1 during bouts of sleep in the first few hours following conditioning was sufficient to disrupt visually-cued fear memory consolidation. Together these data suggest that neurons encoding new associative memories are selectively reactivated during subsequent sleep, and that this sleep reactivation plays a necessary role in memory consolidation.

## What Drives Reactivation/Replay, and How Does It Promote Memory Consolidation?

One plausible explanation for the preferential occurrence of reactivation and sequential replay during sleep states is that sleep oscillations appear to play a critical role in promoting their occurrence. Sequential replay events (involving sequential reactivation of hippocampal place cells active during previous exploration) have been reported to occur in the context of sharp wave-ripple events (present in quiet wake and NREM sleep; >100 Hz) and theta oscillations (present during locomotion in wake and REM sleep; defined as a relatively broad [4–12 Hz] or narrow [6–8 Hz] frequency band). In neocortical networks (e.g., in V1), sequential replay has also been observed (Ji and Wilson, 2007), although the role of thalamocortical oscillations in replay occurrence is still a matter of speculation (Puentes-Mestril et al., 2019). The mechanisms involved in promoting selective reactivation of learning-encoding neurons during subsequent sleep (Clawson et al., 2021) also remain a mystery for now. However, available data from studies of initial memory encoding suggest that neurons’ intrinsic excitability may be increased in the hours following learning. For example, CA1 neurons activated by exploration of a novel context show increased firing rate responses to injected current 5 h later than neighboring non-activated neurons (Cai et al., 2016). CFC leads to long-lasting changes in intrinsic excitability across large populations of CA1 neurons (Moyer et al., 1996; McKay et al., 2009, 2013; Ognjanovski et al., 2014). Similarly, cued fear conditioning leads to increases in the proportion of lateral amygdala neurons with activity-driven CREB phosphorylation, which lasts for several hours after learning (Rashid et al., 2016). In the amygdala, such increases in excitability are both a rate limiting step for incorporation of neurons into engrams during encoding, and for long-term memory storage (Yiu et al., 2014; Rashid et al., 2016).

How do replay and reactivation contribute to long-term memory storage? Recent work from our lab suggests that firing of learning-activated ensembles is sufficient to drive reliable downstream spiking in post-synaptic neurons (Clawson et al., 2021). This post-synaptic firing occurs with a relative phasing that is optimal for spike timing-dependent synaptic potentiation. Recent computational modeling data suggest that in the context of post-learning sleep oscillations, this reactivation will occur rhythmically among neurons activated during prior learning, with phasing (relative to neighboring, post-synaptic neurons) that promotes spike timing-dependent potentiation (Roach et al., 2018). Thus, oscillations prominent during sleep states may convert firing rate-based coding (present during awake learning) into a firing phase-based code which is optimal for promoting spike timing-dependent plasticity (Puentes-Mestril et al., 2019). Support for this idea comes from recent recordings from our lab of neuronal and network activity in CA1 before and after CFC (Ognjanovski et al., 2014, 2017). Over several hours following CFC, network activity in CA1 was characterized by higher-amplitude oscillations in both NREM and REM sleep. At the same time, spike timing relationships between recorded neurons became more consistent and stable after CFC vs. at baseline (Wu et al., 2018). These changes were most dramatic during post-learning NREM and REM sleep, and the degree to which spike timing relationships were stabilized was an excellent predictor of the success of CFM consolidation (Ognjanovski et al., 2014, 2017, 2018). Finally, chemogenetic manipulations leading to disruption of CA1 sleep oscillations prevented CFC-driven stabilization of firing relationships (Ognjanovski et al., 2017), and optogenetically driven oscillations stabilized network activity patterns (Ognjanovski et al., 2018).

While the relationship between spike timing-dependent plasticity in neural circuits and memory consolidation is still a matter of speculation (Puentes-Mestril et al., 2019), available data suggest that cellular mechanisms mediating LTP or LTD are an essential component of memory storage (Stefan et al., 2006; Whitlock et al., 2006; Ge et al., 2010). Synaptic strengthening between engram neurons and their neighbors during sleep may lead to allocation of more neurons into memory traces, which should improve consolidation based on stronger and more robust engrams (Roach et al., 2018; Puentes-Mestril et al., 2019). Synaptic weakening may be critical for segregating different memories to distinct engram populations and for pattern separation (Rao-Ruiz et al., 2019).

## Role of Interneurons in Controlling Sleep Oscillations

Interneurons in both the hippocampus and in thalamocortical circuits play an essential role in regulating sleep oscillations. Within the hippocampus, PV+ interneurons in CA1 are essential for the generation of both theta oscillations (Amilhon et al., 2015; Huh et al., 2016) and ripple oscillations associated with sharp waves (Schlingloff et al., 2014; Gan et al., 2017). As we have recently shown, chemogenetic or state-targeted optogenetic suppression of PV+ interneuron activity in the hours following CFC inhibits learning-induced increases in REM theta and NREM sharp wave-ripple oscillations (Ognjanovski et al., 2017, 2018). Critically, disruption of these oscillations prevents learning-driven stabilization of CA1 neurons’ functional connectivity patterns (i.e., spike timing relationships), in a manner similar to post-learning SD (Ognjanovski et al., 2017, 2018; Xia et al., 2017). Conversely, theta-frequency rhythmic optogenetic activation of PV+ interneurons is sufficient to synchronize CA1 network activity during post-CFC SD, and rescues CFM consolidation from deficits caused by SD (Ognjanovski et al., 2018).

Parvalbumin interneurons in the neocortex also play a role in coordinating sleep oscillations. For example, chemogenetic activation of motor cortex PV+ interneurons suppresses NREM slow wave activity (and other oscillations), but, intriguingly, simultaneously augments REM oscillations (Funk et al., 2017). On the other hand, chemogenetic inhibition of PV+ interneurons in either CA1 or mPFC disrupts temporal coordination of NREM sharp wave-ripple oscillations with thalamocortical sleep spindles (7–15 Hz) (Xia et al., 2017). This coordination of hippocampal and cortical oscillations appears to be an essential component of memory consolidation (Rothschild et al., 2017; Xia et al., 2017), by improving functional communication between hippocampus and neocortex (Cowan et al., 2020). Within layer 2–3 of the neocortex, spiking of PV+ interneurons also appear to coordinate higher-frequency intracortical activity patterns with the troughs of sleep spindle oscillations (Averkin et al., 2016; Khodagholy et al., 2017). Critically, increased temporal coordination of ripple-frequency oscillations, between hippocampus and spatial information-processing regions of neocortex characterizes sleep following training on a spatial memory task.

Beyond this, PV+ interneurons in other brain regions are essential contributors to sleep oscillations present in both the hippocampus and neocortex. For example, generation of spindles in NREM is critically dependent on PV+ interneurons in the thalamic reticular nucleus (Clawson et al., 2016; Fernandez et al., 2018; Thankachan et al., 2019; Bandarabadi et al., 2020), which also appear to play important roles in regulating neocortical gamma (30–100 Hz), delta (1–4 Hz), and slow oscillations (<1 Hz). PV+ interneurons projecting from the medial septum to hippocampal subregions fire with highly specific phasing relative to theta and sharp wave oscillations (Unal et al., 2015; Joshi et al., 2017). This suggests that PV+ GABAergic projections from the septum have the capacity to control sleep-associated hippocampal oscillations. These projections may be the same GABAergic septal projections which play a critical role in driving REM theta oscillations and mediating the effects of REM sleep on memory consolidations (Boyce et al., 2016).

Roles of other interneuron subtypes in regulating specific sleep oscillations have been imputed based on the phasing and occurrence of their firing with respect to those oscillations. Subclasses of hippocampal SST+ interneurons have been implicated in regulation of sleep oscillations based on these data. For example, *in vivo* recordings have demonstrated that some subclasses of SST+ interneurons show generally suppressed firing during sleep relative to wake, and are further suppressed during the occurrence of sharp wave-ripples (Katona et al., 2014). SST+ interneurons throughout the hippocampus profoundly suppress activity in surrounding glutamatergic neurons (Royer et al., 2012; Stefanelli et al., 2016; Delorme et al., 2021) whose activity is critical for generating sharp waves and coordinated “dentate spikes”–bursts of synchronous granule cell activity that is propagated to CA3 to generate NREM sleep sharp waves (Meier et al., 2020). Optogenetic activation of SST+ interneurons in CA1 is sufficient to disrupt spontaneous sharp wave-ripple oscillations (Stark et al., 2014). Therefore, SST+ interneurons may gate dentate spikes and sharp wave-ripple generation and propagation through the hippocampal circuit (Evangelista et al., 2020). As described above, we have recently shown that hippocampal SST+ interneurons are selectively activated during a period of brief SD (Delorme et al., 2021). As described above, this activation is likely due to selective effects of acetylcholine on activating the SST+ population, which in turn suppresses activity in neighboring neurons (Hajos et al., 1998; Jia et al., 2010; Lovett-Barron et al., 2014; Raza et al., 2017; Nichol et al., 2018). Thus, it is plausible that suppression of dentate spikes and sharp waves during active wake (and possibly also during REM, where acetylcholine input to the hippocampus is highest) (Kametani and Kawamura, 1990; Teles-Grilo Ruivo et al., 2017) is mediated by SST+ interneurons.

Are neocortical SST+ interneurons also selectively activated during wake and SD, as they are in the hippocampus? Available data suggest that as is true in hippocampus, neocortical SST+ interneurons are selectively activated by acetylcholine. For example, in mouse somatosensory cortex, cholinergic receptor activation alone is capable of dramatically enhancing excitatory drive onto SST+ interneurons (which is otherwise extremely low) (Urban-Ciecko et al., 2018). Critically, chemogenetic activation of SST+ interneurons in mouse motor cortex leads to augmentation of slow wave activity and cortical theta oscillations (the opposite phenotype to that seen with PV+ interneuron activation). Moreover, this chemogenetic manipulation largely occludes increases in slow waves seen during recovery sleep after a period of SD (Funk et al., 2017). The same study found evidence of increased SST+ interneuron activity (relative to that of surrounding neurons) in the cortex during recovery sleep–suggesting that this mechanism may play a role in homeostatic regulation of slow wave activity after sleep loss. This notion is supported by evidence that nNOS-expressing SST+ interneurons (a subpopulation of the SST+ interneuron population) in particular play a role in promoting homeostatic increases in slow wave activity after SD. Transgenic mice with selective ablation of the nNOS-encoding gene *Nos1* in SST+ interneurons show reductions in delta-frequency slow wave activity in the neocortex at baseline, and a complete loss of homeostatic slow wave increases after SD (Zielinski et al., 2019). This nNOS+ neocortical interneuron population tends to be more active in recovery sleep after SD than during SD itself (Gerashchenko et al., 2008). A second (non-SST+) subtype of neuronal nNOS-expressing neurons has also recently been shown to regulate neocortical sleep oscillations. While SST+ and PV+ neocortical interneurons, like principal neurons, are active during slow wave upstates (Zucca et al., 2017), these nNOS+ interneurons active selectively during downstates (when neighboring neurons are generally quiescent) (Valero et al., 2021).

As mentioned above, interneurons appear to be important not only for coordinating oscillations within neocortical columns or hippocampal subregions, but across the extent of both structures, and even between neocortex and hippocampus. Because the spatial coordination of certain oscillations–such as hippocampal-cortical ripples or neocortical slow waves–is so extensive, it is tempting to speculate about how such synchrony is possible. While coordination via cortico-thalamo-cortical loops appears to play a critical role (Contreras et al., 1996; Timofeev and Steriade, 1996; Durkin et al., 2017), direct coordination of inhibitory networks throughout neocortex by interconnected, cooperative neocortical interneuron networks (Karnani et al., 2016b), by thalamic input (Zucca et al., 2019) and by synchronizing projections from claustrum neurons (Narikiyo et al., 2020) have also been identified as contributors.

## Role of Interneurons in Controlling Replay and Reactivation of Learning-Activated Neuronal Firing Patterns

While the role of interneurons in regulating sleep-associated network oscillations may be critical for memory storage, recent work has highlighted other, additional potential roles for interneurons in the process of consolidation. There is considerable evidence that sleep (and sleep loss) modify excitatory/inhibitory balance in neural circuits. Both electrophysiological (Vyazovskiy et al., 2009; Clawson et al., 2018) and more recently, transcriptomic (Puentes-Mestril et al., 2019) data from the neocortex indicates that brief SD leads to greater activation among FS, PV+ interneurons than among pyramidal neurons. This interpretation has been generally supported by recent calcium imaging of neocortical neurons across sleep-wake transitions. This work has demonstrated that suppression of activity among PV+ and SST+ interneurons during NREM sleep (relative to wake) is much more dramatic than for neighboring pyramidal neurons (Niethard et al., 2016). Critically, this same study found that at the NREM→REM transition, calcium signals for pyramidal neurons and SST+ interneurons decreased still further, while signals for PV+ interneurons increased dramatically. This suggests that the balance between excitatory and inhibitory intracortical signaling is distinct for wake, NREM, and REM.

Our recent work in the hippocampus likewise demonstrates that that excitatory/inhibitory balance is dramatically affected by brain state. Over the course of a few hours of SD, SST+ interneurons in the hippocampus become selectively activated, suppressing activity in surrounding DG granule cells and CA1 and CA3 pyramidal neurons (Delorme et al., 2021). As mentioned earlier, this same gating mechanism controls the initial size of engram populations in the hippocampus during encoding (Stefanelli et al., 2016). This scenario may be different from the changes in SST+ and pyramidal neurons in the neocortex, which is consistent with another recent study from our lab, showing that across SD, cell type-specific IEG expression differs between hippocampus and neocortex (Delorme et al., 2019; Puentes-Mestril et al., 2021).

The roles that these changes play in promoting sleep-dependent engram neuron reactivation, sequential replay, and memory consolidation are still unclear. In the *ex vivo* and *in vivo* neocortex, SST+ interneuron activation during rhythmic upstates plays a significant role in constraining pyramidal neuron firing (Neske and Connors, 2016; Naka et al., 2019). In the context of awake behavior, the activity of SST+ (and at least a subset of PV+) interneurons is dynamically regulated by interneuron-targeting, VIP+ interneurons. By inhibiting the activity of interneuron populations which constrain the firing of surrounding pyramidal neurons, in a layer- and column-specific manner, it is thought that VIP+ interneurons provide windows of disinhibition which are critical for learning and synaptic plasticity (Pi et al., 2013; Karnani et al., 2016a). Available data suggest that a similar VIP+ interneuron-mediated disinhibitory circuit motif gates PV+ interneuron activity and plasticity associated with contextual and goal-orientated spatial learning in the hippocampus, and with rotarod training in the motor cortex (Donato et al., 2013; Turi et al., 2019). Regulation of interneurons by this mechanism appears to be involved in learning-associated sequencing of neuronal activities in the context of learning. For example, during learning of a coordinated running task, pyramidal neurons in mouse motor cortex show compression of their sequential firing locked to executing the learned movement; this compression of sequential activity (which relies on LTP-like mechanisms) is associated with improved motor performance (Adler et al., 2019). Critically, this process is mediated by activation of VIP+ interneurons in the motor cortical network, and downstream suppression of SST+ interneurons. Similarly, VIP+ interneurons in the prefrontal cortex appear to facilitate performance on memory-guided tasks (such as a Go/No-Go sensory discrimination task) by suppression of activity in surrounding PV+ and SST+ interneurons (Kamigaki and Dan, 2017). Much less is known about the regulation of VIP neurons as a function of behavioral state. One intriguing recent finding suggests that VIP+ interneuron activation levels are tightly regulated by behavioral states and associated oscillations. The authors found that VIP+ interneurons are selectively activated in the hippocampus during theta oscillations, but have suppressed activity during sharp wave-ripple events (Lui et al., 2020). However, these calcium signal recordings took place during wakefulness–it is unclear how VIP+ interneuron activity is regulated in the context of these oscillations in REM and NREM sleep. Another unanswered question is the extent to which sequential neuron activation (post-learning reactivation of engram neurons) during sleep-dependent consolidation is associated with VIP+ interneuron-mediated disinhibition in neocortical neurons.

A few recent studies have focused on how regulation of neocortical inhibitory circuits could promote synaptic plasticity in the context of sleep oscillations, using *in vivo* calcium imaging. One of these characterized the relative activation of PV+ interneurons, SST+ interneurons, and pyramidal cells in the context of NREM slow oscillations and spindles (Niethard et al., 2018). The authors found that during isolated slow oscillations and isolated spindles, activity in pyramidal neurons was suppressed by dendrite-targeted inhibition from SST+ interneurons and cell body-targeted inhibition from PV+ interneurons, respectively. However, when spindles and slow oscillations occurred simultaneously, SST+ interneuron inhibition of dendrites was suppressed, PV+ interneuron somatic inhibition was enhanced, and pyramidal cell excitatory drive was dramatically enhanced. This finding is consistent with the recent report of enhanced calcium influx to neocortical pyramidal neurons’ dendrites during NREM spindle oscillations (Seibt et al., 2017). This enhancement is not only reflected as an increase in the frequency of calcium transients, but also as increased synchrony of calcium transients between individual dendritic branches of neurons in the same neocortical region. Still, another recent study found higher-frequency, but asynchronous, dendritic calcium transients during REM sleep among neocortical pyramidal neurons in primary motor cortex (M1) in the context of motor learning (Li et al., 2017). This asynchronous dendritic calcium influx through NMDA receptors was essential for both learning-driven synaptic elimination and synaptic strengthening. Thus, differential gating of dendritic vs. somatic calcium via dendrite- and soma-targeting interneurons may play a critical role in sleep-dependent synaptic plasticity in the context of memory consolidation.

While it is unclear how these mechanisms function in the context of hippocampus-dependent memory consolidation, it is very clear that SST+ interneurons have reduced activity during sleep vs. wake, and that this plays a critical role in memory storage during sleep (Delorme et al., 2021). Future studies will be needed to clarify how dendritic vs. somatic calcium influx varies as a function of brain state in hippocampal granule cells and pyramidal neurons, and how this affects memory storage.

## Brain State-Regulated Interneuron Functions in Brain Disorders

Simultaneous disruptions in both sleep behavior and cognition have been reported in neurodevelopmental disorders such as schizophrenia, depression, and dementia. For all of these disorders, the underlying neuropathology is only (at best) partially understood, and a better understanding of these mechanisms would profoundly impact the targeting of therapeutic strategies. Critically, impaired functioning of GABAergic interneurons has been implicated in several neurodegenerative and neuropsychiatric disorders (Ruden et al., 2021; Song et al., 2021).

Alzheimer’s disease (AD) is a progressive neurodegenerative disorder, and one of the most common causes of dementia. Although the underlying mechanisms of AD are still not completely understood, disrupted interneuron function has been associated with AD (Reid et al., 2021). For example, accumulation of the amyloid-β (Aβ) protein, which is a classical hallmark of AD pathology, has been shown to alter excitatory/inhibitory balance; this aspect of disease pathology is implicated in the observed learning and memory deficits associated with AD (Verret et al., 2012; Palop and Mucke, 2016; Hijazi et al., 2020). Recent work in AD mouse models has found either decreased, unchanged, or increased PV+ cell density in CA1 (Hollnagel et al., 2019; Reid et al., 2021). The differences in findings are likely explained in part by differences between various transgenic AD mouse models and the age at which mice are examined in different studies (Ruden et al., 2021). Recent studies have more consistently found reductions in DG PV+ expression in older AD transgenic animals compared to their wild-type counterparts (Loreth et al., 2012; Reid et al., 2021). SST+ interneurons’ immunoreactivity is also altered in both AD humans and animal models. For example, multiple studies, in both animal models and human post-mortem brains, found decreased somatostatin expression in CA1, but no clear change in CA3 or DG (Reid et al., 2021). Somatostatin expression is also reduced in the human neocortex in early AD (Guennewig et al., 2021). To address the causal role for such changes in neurocognitive phenotypes associated with AD, various interneuron-targeted strategies have been deployed in mouse models in an attempt to rescue AD-related pathology. Some of these experimental strategies have met with success. For example, transplanting interneuron progenitor cells into the hippocampus of AD mice prevents deficits in learning and memory, rescues impairments in synaptic plasticity, and reduces neuronal hyperexcitability (Lu et al., 2020). Another recent study noted PV+ interneuron hyperexictability is an early neuropathological feature in APP/PS1 mice, and showed that chemogenetic inhibition of PV+ interneurons restored excitatory/inhibitory balance and achieved a long-term rescue of hippocampal network and memory deficits, along with reductions in amyloid plaque deposition (Hijazi et al., 2020). Critically, AD is characterized by changes to interneuron-regulated sleep oscillations such as NREM slow waves and spindles, which are an excellent predictor of underlying AD neuropathology (Kam et al., 2019; Winer et al., 2019; Caccavano et al., 2020; Prince et al., 2021; Zhen et al., 2021). This phenomenon is also seen in mouse models of AD. For example, in three recent studies using APP/PS1 and 3xTg-AD mice (Zhurakovskaya et al., 2019; Benthem et al., 2020; Prince et al., 2021), the occurrence of NREM hippocampal sharp wave-ripples was significantly decreased, as was coupling of oscillations (e.g., sharp waves and slow waves, spindles and slow waves) between hippocampus and cortex. In 5xFAD mice, which typically show a more progressive and severe AD phenotype, altered frequency and amplitude of hippocampal sharp wave-ripples are accompanied by selective reductions of PV+ basket cells’ activity during these oscillations, and corresponding aberrant increases in pyramidal neuron firing (Caccavano et al., 2020). In support of the idea that interneuron regulation of these oscillations may be a critical mediating factor in AD, a recent study demonstrated that rhythmic optogenetic PV+ or SST+ interneuron activation in Aβ-treated hippocampal slices restored Aβ-induced disruptions of hippocampal network oscillations and oscillation-induced LTP, respectively (Park et al., 2020). Altogether, these studies indicate that dysfunction of PV+ and SST+ interneurons may play an important role in the development of AD pathology, and that dysregulation of sleep oscillations may play a critical role. Thus, therapeutics targeting interneurons may benefit AD-induced disruption of brain plasticity, sleep oscillation, and learning and memory.

Attenuated sleep oscillations, altered sleep patterns, and dysfunctional PV+ and SST+ GABAergic interneurons have also been observed in schizophrenia (Lewis and Sweet, 2009), which is a multifaceted mental disorder characterized by cognitive deficits. NREM sleep spindle disruption and suppression is a highly consistent finding in schizophrenic patients, and has predictive value of cognitive, positive, and negative symptoms (Kaskie et al., 2019; Au and Harvey, 2020; Gerstenberg et al., 2020; Markovic et al., 2020; Zhang et al., 2020). This selective disruption of sleep spindles is linked to loss of PV+ interneurons in the thalamic reticular nucleus of schizophrenic patients (Steullet et al., 2018). Loss of both PV+ and SST+ interneurons has also been reported in post-mortem hippocampus (Konradi et al., 2011) and neocortex (Gonzalez-Burgos et al., 2015; Dienel and Lewis, 2019) of patients with schizophrenia. For example, somatostatin immunoreactivity is significantly lower, and PV+ interneuron immunoreactivity shows a tendency for decrease, in post-mortem hippocampal tissue from schizophrenic patients compared with controls (Konradi et al., 2011). Additionally, recent genetic data suggest that dysregulation of inhibitory synapses is a critical functional feature of schizophrenia. For example, rescue of disrupted *Npas4* expression to the prefrontal cortex of mice with schizophrenia-associated 16p11.2 microduplications led to a rescue of neuronal excitatory/inhibitory balance, and behavioral phenotypes, in this mouse model (Rein et al., 2020). Moreover, suppression of *Npas4* expression in PV+ interneurons phenocopies behavioral deficits seen in developmental mouse models of schizophrenia (Shepard et al., 2019). Critically, sleep-dependent memory processing appears to be adversely affected in schizophrenia (Manoach et al., 2010; Wamsley et al., 2012; Genzel et al., 2015). While multiple studies have aimed to rescue disrupted overnight memory consolidation and other symptoms using hypnotic drugs which restore some features of sleep oscillations, to date they have met with limited success (Tek et al., 2014; Kishi et al., 2017; Mylonas et al., 2020). For example, a recent randomized clinical trial using eszopiclone found that patients’ (and controls’) sleep spindle density was enhanced by the hypnotic, but sleep-dependent memory consolidation was not enhanced in either group (Mylonas et al., 2020). Clearly, a better understanding of the role of interneurons in promoting memory consolidation during sleep, and of the effects of hypnotic drugs on brain plasticity (Seibt et al., 2008; Aton et al., 2009a), are needed to inform therapeutics for schizophrenia.

Major depressive disorder (MDD) is another multifaceted condition, typically characterized by low mood, anhedonia, and cognitive deficits including attention and memory problems (Anderson et al., 2020). Sleep disruption has long been described as a characteristic of depression, and recent studies of patients have found enhanced slow wave homeostasis (Frey et al., 2012), disrupted NREM sleep spindles (Lopez et al., 2010; Nishida et al., 2016), and impaired sleep-dependent memory consolidation (Dresler et al., 2010, 2011; Genzel et al., 2011, 2015; Nishida et al., 2016) in MDD patients. Disturbances in the excitatory/inhibitory balance in corticolimbic brain structures have been observed in depression (Thompson et al., 2015). SST+ interneurons in particular are thought to play an important role in the onset of depression, as somatostatin immunoreactivity is decreased in the dorsolateral prefrontal cortex, amygdala, and anterior cingulate cortex post-mortem tissue from depressed patients (while PV+ interneurons appear to be unaffected) (Rajkowska et al., 2007; Tripp et al., 2011; Douillard-Guilloux et al., 2017; Fee et al., 2017; Anderson et al., 2020). How these differences relate to the symptomatology of depression is an area of active investigation. However, in a mouse model of depression, increasing SST+ interneurons’ excitability via cell type-specific knockout the γ2 GABA receptor subunit reduces anxiety- and depression-related behaviors (Fuchs et al., 2017). Mice with this genetic manipulation spent more time in the open arms of the elevated plus maze, reduced time to feed in a novel environment, and less time spending immobile in the forced swim test (Fuchs et al., 2017). This suggests that augmenting the function of SST+ interneurons may be sufficient to rescue depression-like behaviors.

Altogether, the available data suggest that interneurons likely play an important role in the development or progression of many psychiatric disorders, and that these may be mediated in part through effects on sleep oscillations and sleep-dependent circuit plasticity.

## Conclusion and Future Directions

Available data suggest that interneurons play vital roles in gating and timing the activation of engram neurons in the context of memory encoding. Our recent data suggest that they likely play similar roles during the process of memory consolidation–either permitting or suppressing reactivation of these memory-encoding neurons during subsequent sleep. Beyond this, PV+ and SST+ interneurons regulate sleep oscillations that play a vital role in sleep-dependent memory consolidation. Recent data from our lab and others indicated that hippocampal memory consolidation is highly sensitive to post-learning manipulations of interneuron activity. Future studies will be needed to clarify what aspects of NREM and REM sleep regulate the seemingly distinct roles PV+ and SST+ in consolidation, as well as how interneuron populations contribute to communication across various brain structures during sleep. Because interneurons are so functionally and structurally diverse, a better understanding of their subtypes and interactions across brain states is also needed. Finally, it will be important to understand how biological factors such as age, sex, and stress–which can alter both sleep and cognition–contribute to interneuron-mediated regulation of memory processing. An understanding of these mechanisms will have broader implications for our ability to diagnose and treat neuropsychiatric disorders which are associated with both sleep and cognitive disruption, as well as underlying pathological changes to interneurons.

## Author Contributions

Both authors listed have made a substantial, direct and intellectual contribution to the work, and approved it for publication.

## Conflict of Interest

The authors declare that the research was conducted in the absence of any commercial or financial relationships that could be construed as a potential conflict of interest.

## Publisher’s Note

All claims expressed in this article are solely those of the authors and do not necessarily represent those of their affiliated organizations, or those of the publisher, the editors and the reviewers. Any product that may be evaluated in this article, or claim that may be made by its manufacturer, is not guaranteed or endorsed by the publisher.

## Abbreviations

AD: Alzheimer’s disease
A β: amyloid- β
BDNF: brain-derived neurotrophic factor
CA: cornu ammonis
CCK: cholecystokinin
CFC: contextual fear conditioning
CFM: contextual fear memory
ChR2: channelrhodopsin
CSEA: cell type-specific enrichment analysis
DG: dentate gyrus
FS: fast-spiking
GABA: gamma aminobutyric acid
IEG: immediate-early gene
LTP: long-term potentiation
LTD: long-term depression
M1: primary motor cortex
MDD: major depressive disorder
mPFC: medial prefrontal cortex
mRNA: messenger ribonucleic acid
NMDA: *N*-methyl -D-aspartate
nNOS: neuronal nitric oxide synthase
NPY: neuropeptide Y
NREM: non-rapid eye movement
PC: principal (glutamatergic) celll
PV: parvalbumin
REM: rapid eye movement
SD: sleep deprivation
SO: stratum oriens
SP: stratum pyramidale
SR: stratum radiatum
SST: somatostatin
TRAPs: targeted recombination in activated populations
V1: primary visual cortex
VIP: vasoactive intestinal peptide
WGCNA: weighted gene co-expression network analysis.
